# Liprotides kill cancer cells by disrupting the plasma membrane

**DOI:** 10.1038/s41598-017-15003-6

**Published:** 2017-11-09

**Authors:** Henriette S. Frislev, Theresa Louise Boye, Jesper Nylandsted, Daniel Otzen

**Affiliations:** 1 Interdisciplinary Nanoscience Center (iNANO), Department of Molecular Biology and Genetics, Aarhus University, Gustav Wieds Vej 14, DK-8000 Aarhus, Denmark; 2 Membrane Integrity Group, Cell Death and Metabolism Unit, Danish Cancer Society Research Center, Strandboulevarden 49, DK-2100 Copenhagen, Denmark

## Abstract

HAMLET (human α-lactalbumin made lethal to tumour cells) is a complex of α-lactalbumin (aLA) and oleic acid (OA) which kills transformed cells, while leaving fully differentiated cells largely unaffected. Other protein-lipid complexes show similar anti-cancer potential. We call such complexes *liprotides*. The cellular impact of liprotides, while intensely investigated, remains unresolved. To address this, we report on the cell-killing mechanisms of liprotides prepared by incubating aLA with OA for 1 h at 20 or 80 °C (lip20 and lip80, respectively). The liprotides showed similar cytotoxicity against MCF7 cells, though lip80 acts more slowly, possibly due to intermolecular disulphide bonds formed during preparation. Liprotides are known to increase the fluidity of a membrane and transfer OA to vesicles, prompting us to focus on the effect of liprotides on the cell membrane. Extracellular Ca^2+^ influx is important for activation of the plasma membrane repair system, and we found that removal of Ca^2+^ from the medium enhanced the liprotides’ killing effect. Liprotide cytotoxicity was also increased by knockdown of Annexin A6 (ANXA6), a protein involved in plasma membrane repair. We conclude that MCF7 cells counteract liprotide-induced membrane permeabilization by activating their plasma membrane repair system, which is triggered by extracellular Ca^2+^ and involves ANXA6.

## Introduction

α-lactalbumin (aLA) is an acidic calcium metallo-protein (14.2 kDa, pI 4–5) present in the milk of almost all animal species^1–3^, and is involved in the formation of lactose in mammary secretory cells during lactation^4,5^. In 1995 Svanborg and coworkers discovered that aLA in complex with oleic acid (OA) was able to kill cancer cells and named the complex HAMLET (human alpha-lactalbumin made lethal to tumour cells)^6–8^. OA is a sparingly soluble monounsaturated omega-9 fatty acid (18:1 cis-9) and there is mounting evidence that HAMLET’s cytotoxicity can be ascribed to OA, while the function of aLA is to keep OA in solution and transport it to its site of action^9–12^. Nevertheless, the mechanism(s) of HAMLET-induced cell death remain unclear. Suggestions include apoptotic-like cell death and permeabilization of lysosomes^13–15^, but HAMLET also interacts with many different cell components including the plasma membrane, proteasomes, mitochondria, histones and chromatin^14,16–19^. Growing interest in this phenomenon has led to the preparation of cytotoxic HAMLET-like complexes formed by different proteins, fatty acids and methods. aLA can be substituted with other proteins with no effect on the cytotoxicity, but replacement of OA dramatically reduces cytotoxicity^9,18–21^. While it is difficult for aLA to form complexes with saturated fatty acids, OA can more easily be replaced by other unsaturated fatty acids provided the fatty acids contain *cis* double bonds, although this reduces cytotoxicity in some cases to the level of untreated cells^20–22^^;^. A variety of different preparation methods can be used to form HAMLET-like complexes^23–27^ and a common requirement seems to be partial protein unfolding which facilitates fatty acid binding, leading to what we call a *liprotide* (complex of lipids and partially unfolded proteins).

Small-angle X-ray scattering (SAXS) data indicate that liprotides have a micelle-like core of fatty acids decorated with a shell of partially denatured protein, known as the core-shell model^22,28^. Core-shell liprotides have a diameter of ~100 Å and are proposed to have a central core of 12–33 fatty acids surrounded by 2–4 partially unfolded protein molecules^22,28^. At higher OA:aLA ratios, a species known as the multi-shell state is formed, consisting of a central core-shell liprotide decorated with a shell of OA and an outer shell of aLA^22^.

Liprotides can transfer the fatty acid component from liprotides to vesicles, resulting in release of monomeric and at least partially refolded aLA and increased membrane fluidity^22,29^. It remains to be determined if fatty acid transfer is directly connected to the cell death mechanism. The primary function of the plasma membrane is to separate the intra- and extracellular environments. Consequently, disrupting the plasma membrane can destroy this compartmentalization leading to cell death^30^, if the cell is not able to repair the damage. The plasma membrane repair system is triggered by Ca^2+^ influx through a membrane hole^31^. A family of 12 proteins named annexins (ANXA1-ANXA11 and ANXA13) functions as Ca^2+^ sensors, and some are important components in the plasma membrane repair system. They are structurally related but each annexin requires different free Ca^2+^ concentrations for their activation and prefers different interaction partners^30,32,33^. Binding of Ca^2+^ to annexins induces a conformational change, which enables them to interact with negatively charged phospholipids in membranes. This allows them to promote membrane segregation, vesicle trafficking, vesicle fusion, cytoskeletal depolymerisation and membrane reorganization^34,35^. Importantly, ANXA1, ANXA2, ANXA5 and ANXA6 collaborate in a complex network to reseal a torn membrane^36–38^. To this end, cancer cells experience increased membrane lesions due to intrinsic metabolic stress and when navigating through the extracellular matrix but appear to compensate with upregulated annexin expression^35,39^.

Here, we focus on the cellular and membrane impact of liprotides formed at 20 °C (lip20) and 80 °C (lip80). Lip80 only differ from lip20 by having intermolecular disulphide bonds between aLA molecules, which increases its resistance to refolding in the presence of Ca^2+^ ^22,40^. We examined the anti-cancer potential of liprotides by treating MCF7 cells (human breast adenocarcinoma cell line) with lip20 and lip80 in the presence or absence of Ca^2+^ to address the role of the cell membrane repair system. We investigate this aspect further by silencing the plasma membrane repair protein, ANXA6. We provide evidence that liprotides trigger cell death by inducing plasma membrane permeabilization, which cells attempt to counteract by activating their cell membrane repair system.

## Results

### Liprotides prepared at 20 °C kill cancer cells faster than liprotides prepared at 80 °C

**Figure 1:**
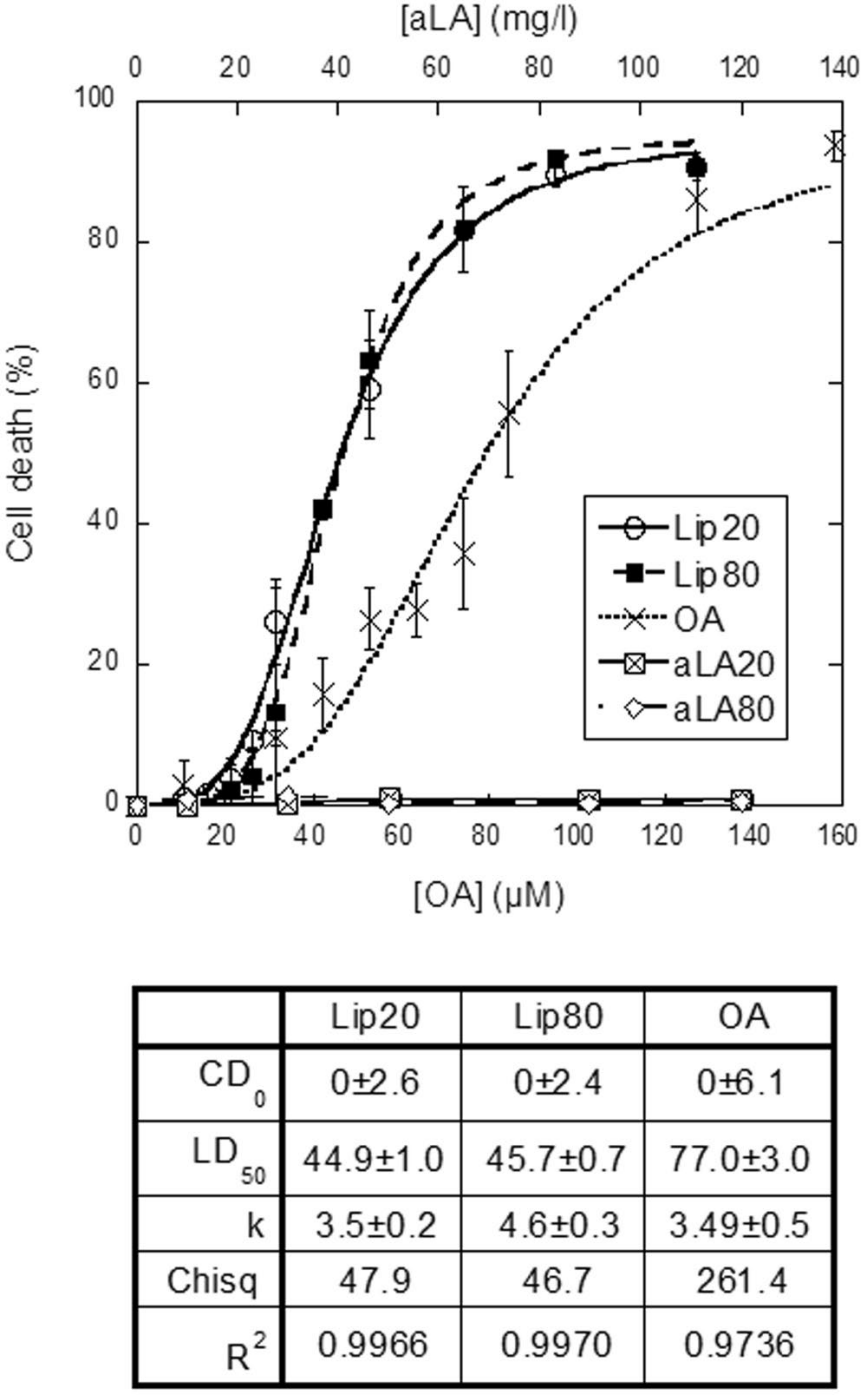
Cell death induced by lip20, lip80, OA, aLA20 and aLA80. The percentage dead cells are plotted versus OA concentration for liprotides and OA, and as function of aLA concentration for aLA20 and aLA80. A sigmoidal curve (Eq. 1) was fitted to data for lip20, lip80 and OA, with fitted parameters indicated below the figure.

We prepared liprotides at two different temperatures, lip20 and lip80, and analysed their ability to kill cancer cells, using the MCF7 breast carcinoma cell line as model. Due to their differences in preparation, lip20 and lip80 are expected to differ in their disulphide bonding pattern. Natively folded aLA has four disulphide bonds, which can be shuffled at elevated temperatures. We have previously demonstrated that the four disulphide bonds in lip20 are intramolecular as in the native state, whereas lip80 contains several intermolecular disulphide bonds^22^. These intermolecular disulphide bonds do not change the overall liprotide core-shell structure^22,40^ but might affect liprotide cytotoxicity. MCF7 cells were exposed to different concentrations of lip20 and lip80, leading to a sigmoidal dose-response curve when cell death (%) was plotted as function of the OA concentration (Fig. 1). The two DNA binding fluorophores Hoechst-33342 and propidium iodide (PI) were used to stain all cells and dead cells respectively. In both cases, DNA binding leads to an increase in fluorescence. Hoechst-33342 is cell permeable and can therefore be used to determine total cell number, while PI is plasma membrane impermeable and therefore only binds to dead (permeable) cells. By fitting a sigmoidal curve (Eq. 1) to the data, we obtained the dose causing 50% cell death (LD_50_), which was 44.9 ± 1.0 and 45.7 ± 0.7 μM for cells treated with lip20 and lip80, respectively. Thus the two liprotides have essentially identical anti-cancer potential. In contrast, aLA20 (aLA incubated at 20 °C, 1 h) and aLA80 (aLA incubated at 80 °C, 1 h) alone failed to induce any cell death, and the OA component alone only had an effect at higher concentrations (LD_50_ = 77.0 ± 3.0 μM). This strongly emphasizes the role of liprotides in mobilizing and delivering OA. Liprotide cytotoxicity was not limited to the MCF7 breast carcinoma cell line (positive estrogen (ER+) and progesterone (PR+) receptor and negative human epidermal growth factor (HER2-) receptors); both liprotides kill MDA-MB-231 (triple negative breast cancer cells; ER-/PR-/HER2-) and HeLa cells (human cervical epithelia adenocarcinoma) with efficiency comparable to that against MCF7 cells (Supplementary Fig. S1). The effect on the MDA-MB-231 cells also illustrates that liprotides kill breast cancer cells independent of ER, PR and HER2 status.

**Figure 2:**
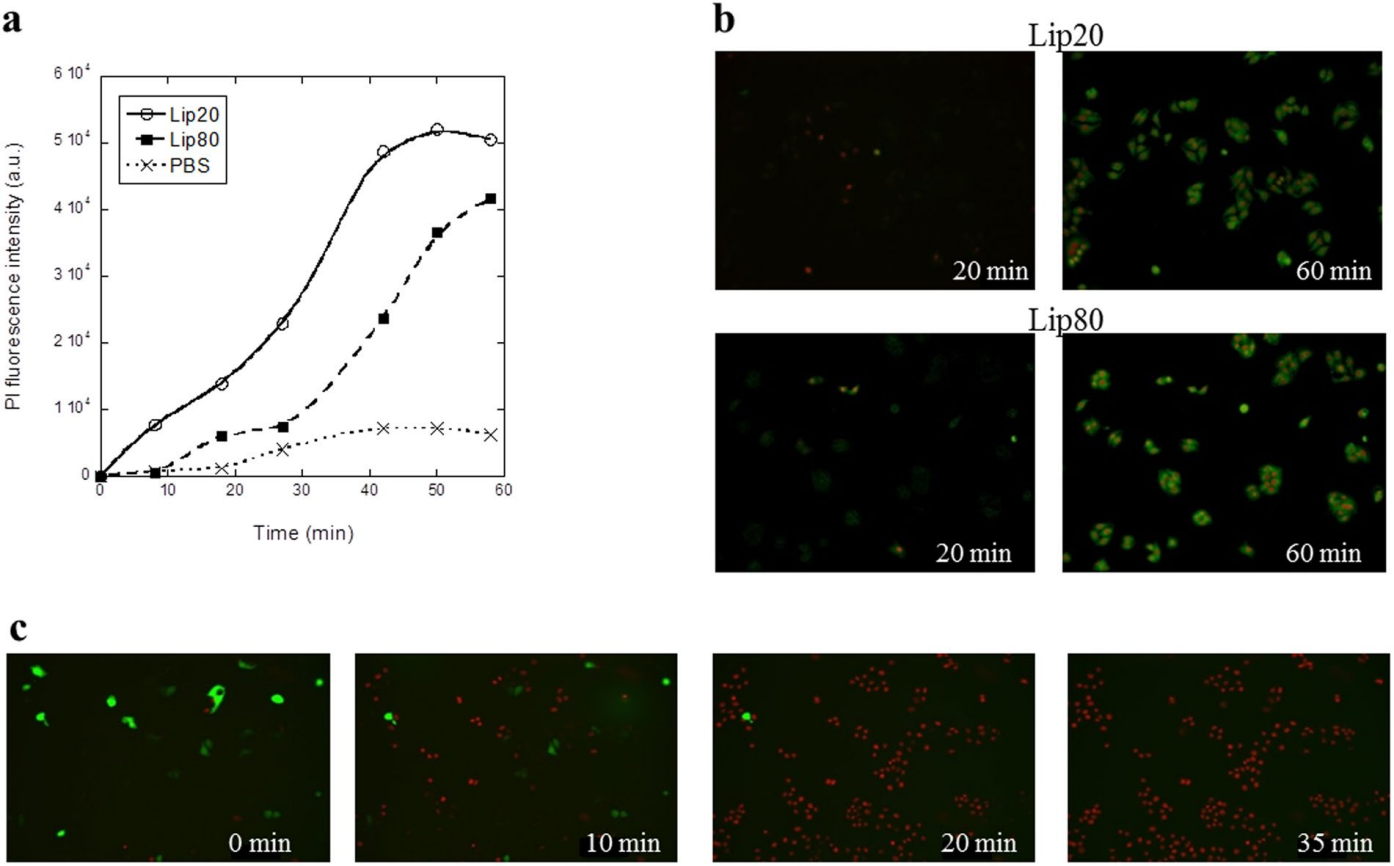
Rate of plasma membrane permeabilization induced by lip20 and lip80. (**a**) The integrated PI fluorescence intensity plotted as function of treatment time for 42 µM lip20, 42 µM lip80 and PBS as control. (**b**) Images of cells after 20 min and 60 min of treatment with 42 µM lip20 and 42 µM lip80. FM1–43 in green and impermeable Hoechst in red. (**c**) Release of cytoplasmic GFP induced by lip80. Images are shown after 0 min, 10 min, 20 min and 35 min of incubation with 212 µM lip80. GFP in green and impermeable Hoechst in red.

Next, the kinetics of the breakdown of membrane integrity induced by liprotides, measured through PI binding, was investigated as a potential proxy for cell death. Figure 2a shows the progression of the integrated PI fluorescence over time after treatment with lip20, lip80 and PBS. While both liprotides are more effective than PBS alone, lip20 give rise to a significantly higher fluorescence than lip80 at all time points, indicating that cells are permeabilized faster by lip20. Membrane integrity and cell death induced by lip20 and lip80 were also visualized by fluorescence microscopy using the plasma membrane binding dye FM1–43 as well as the essentially impermeable Hoechst-33258 (Fig. 2b; Supplementary Videos S1 and S2). The imaging experiments confirmed that cells are permeabilized by lip20 and lip80, resulting in intense nuclear Hoechst-33258 staining as well as cytoplasmic FM1–43 staining. Moreover, time-lapse imaging of MCF7 cells overexpressing green fluorescence protein (GFP) showed that cells were rapidly permeabilized, resulting in cell membrane puncture and release of cytoplasmic GFP to the extracellular environment (Fig. 2c; Supplementary Video S3). Overall, these results indicate that both liprotides kill cancer cells though lip20 works more rapidly than lip80, despite the similar concentration of OA in the two liprotides. One reason for this difference could be intermolecular disulphide bonds in lip80 which either lead to slower release of OA or modulate the mechanism of binding of liprotides to cells. This is addressed further below using crosslinked liprotides.

### Ca^2+^ depletion and ANXA6 knockdown make cancer cells more sensitive to liprotides

**Figure 3:**
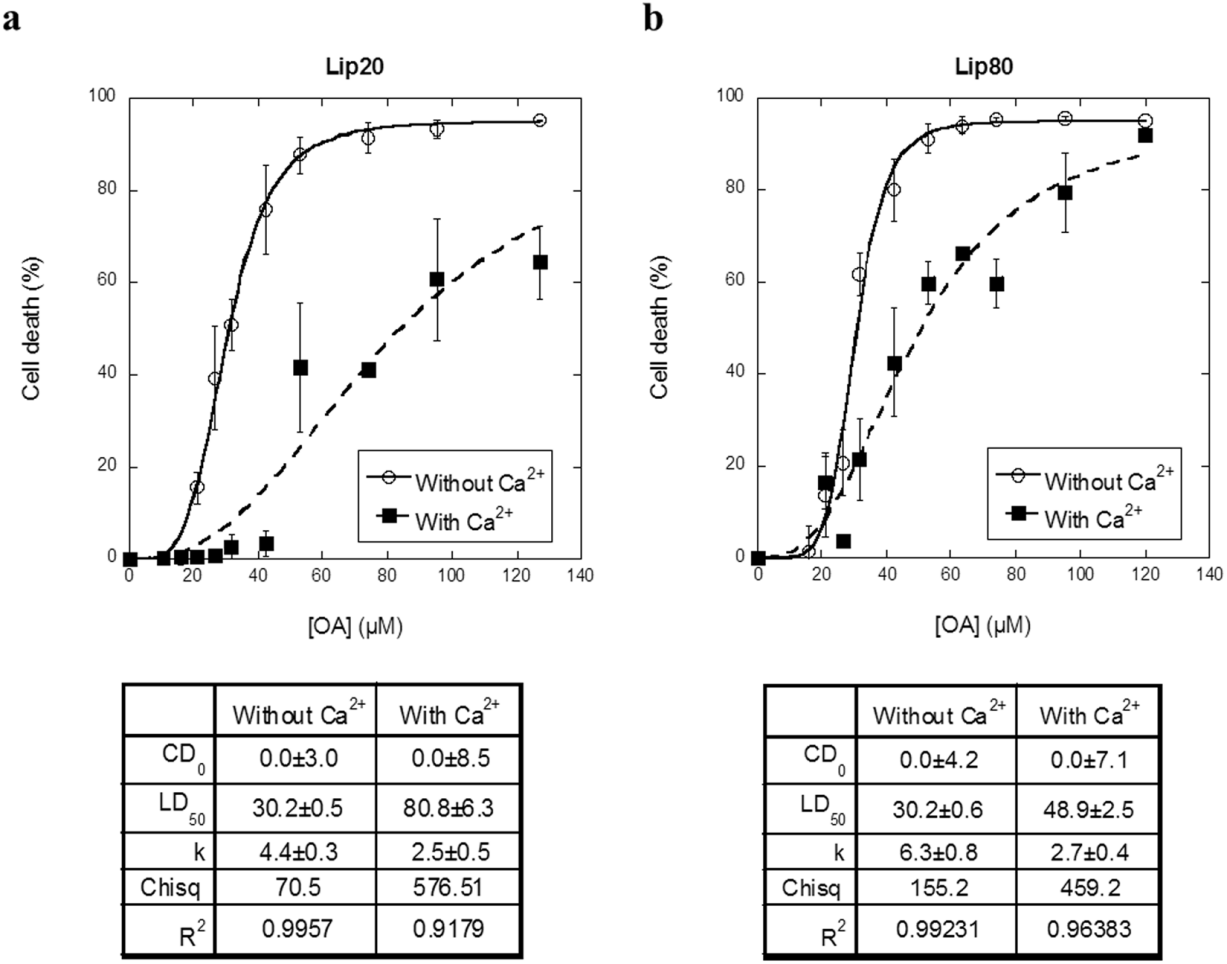
The effect of removing Ca^2+^ from the cell media. (**a**) Cells treated with lip20 in media without and with Ca^2+^. (**b**) Cells treated with lip80 in media with and without Ca^2+^. Data fitted as in Fig. 1 and fitted parameters indicated below the figures.

**Figure 4:**
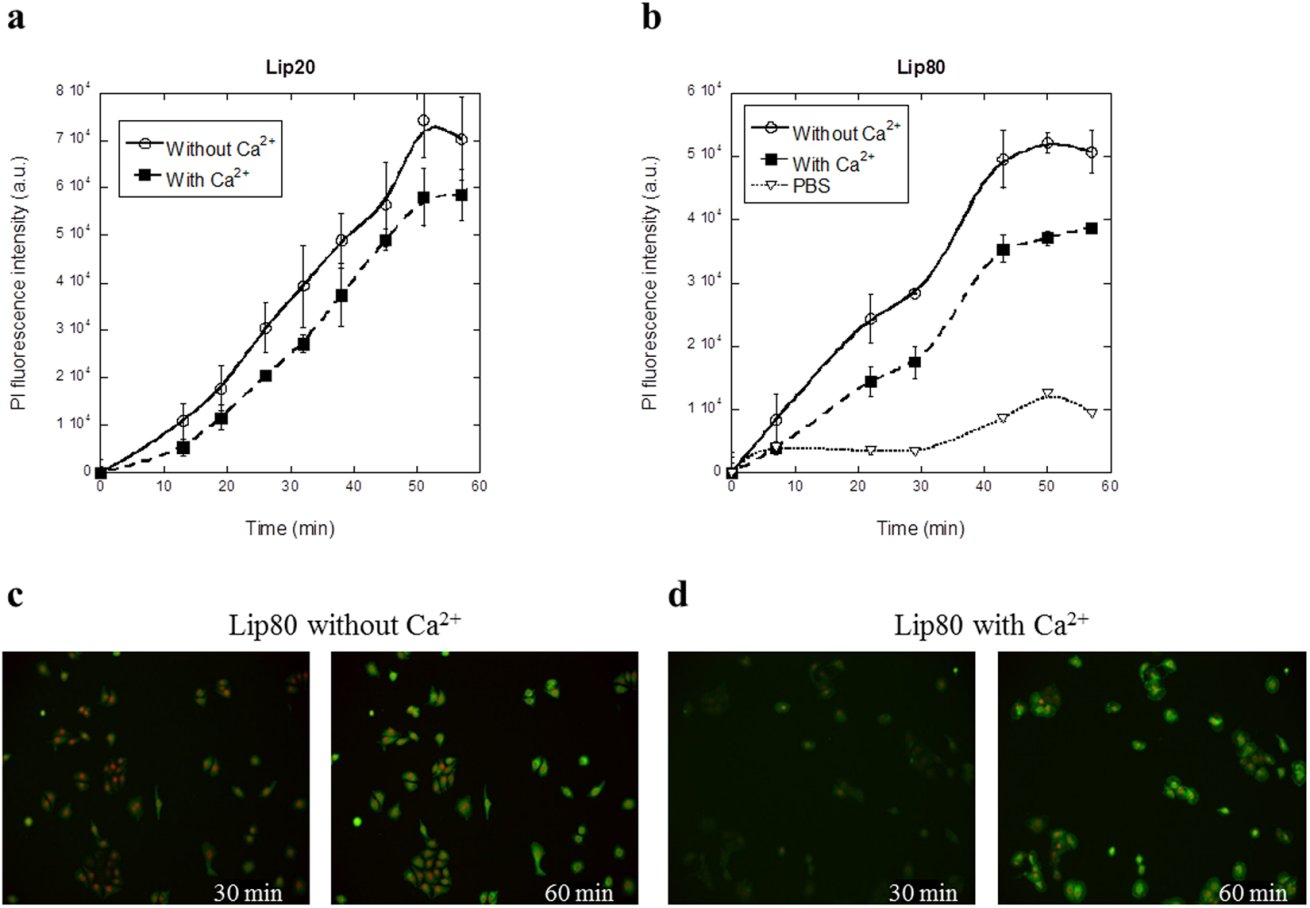
Rate of plasma membrane permeabilization induced by lip20 and lip80 in presence and absence of Ca^2+^. The integrated PI fluorescence intensity plotted as a function of treatment time. (**a**) Cells treated with 52 µM lip20 in media without and with Ca^2+^. (**b**) Cells treated with 52 µM lip80 and PBS in media without and with Ca^2+^. (**c**) Images of cells after 30 min and 60 min of treatment with 42 µM lip20. (**d**) Images of cells after 30 and 60 min of treatment with 42 µM lip80. FM1–43 in green and impermeable Hoechst in red.

The phospholipid plasma membrane provides a barrier between the intracellular and extracellular environment. Membrane damage can compromise this barrier and thus lead to uncontrolled flux of small molecules across the membrane. The concentration of cytosolic Ca^2+^ is 1000-fold lower than its extracellular counterpart, and Ca^2+^ influx initiated by a disruption of the plasma membrane will initiate the plasma membrane repair system^30,31^. The proteins involved in the repair system are either Ca^2+^ sensors by themselves or are functionally regulated by Ca^2+^. We have previously reported that transfer of the fatty acid component in liprotides to vesicles increases membrane fluidity^12,22^. We therefore decided to investigate whether liprotides’ ability to kill cancer cells is due to disruption of the plasma membrane and if they can counteract this effect by activating their membrane repair system. Our first step was to investigate the effect of Ca^2+^. Lip20 and lip80 were added to cells in media with or without Ca^2+^ and the cell death was measured by a PI exclusion assay. For lip20, the LD_50_ value is 80.8 ± 6.3 μM in the presence of Ca^2+^ as compared to 30.2 ± 0.5 in its absence (Fig. 3a). For lip80, the corresponding values are 48.9 ± 2.5 µM and 30.2 ± 0.6 μM (Fig. 3b). Thus, removing Ca^2+^ from the media dramatically decreases the LD_50_ value for both lip20 and lip80, indicating that Ca^2+^ is required for cells to counteract plasma membrane permeabilization induced by liprotides. It should be noted that aLA has a high-affinity Ca^2+^ binding site and that removal of Ca^2+^ is a prerequisite for formation of the aLA-fatty acid complex. Therefore Ca^2+^ could in principle bind to and modify liprotides. In fact, we have previously shown that addition of several-fold excess Ca^2+^ to lip20 leads to complete refolding of aLA and breakdown of the liprotide structure, whereas there is no effect on aLA structure in lip80^22,40^. Although the low Ca^2+^ stability of lip20 complicates data analysis, it is worth noting that lip80, which is not Ca^2+^-sensitive itself, is also more potent as cytotoxin in the absence of Ca^2+^. We interpret these results to mean that liprotides disrupt the plasma membrane and activate the Ca^2+^ dependent plasma membrane repair system; the greater reduction in lip20 cytotoxicity in the presence of Ca^2+^ (compared to lip80) can be attributed to the double effect of partial breakdown of lip20 in combination with activation of the plasma repair system. Measurements over time indicate that membrane permeabilization is slowed down by the presence of Ca^2+^ (Fig. 4a and b), particularly for lip80; the standard deviations of the measurements with lip20 are too large to conclude that the effect of Ca^2+^ is statistically significant although PI entry levels are consistently lower in the presence of Ca^2+^ also for this liprotide. Cell death and membrane integrity induced by lip80 were also visualized by fluorescence microscopy using the plasma membrane binding dye FM1–43 as well as impermeable Hoechst-33258 (Fig. 4c and d; Supplementary Videos S4 and S5). The imaging experiments confirmed that cells are permeabilized by lip80, resulting in intense nuclear Hoechst-33258 staining as well as cytoplasmic FM1–43 staining.

**Figure 5:**
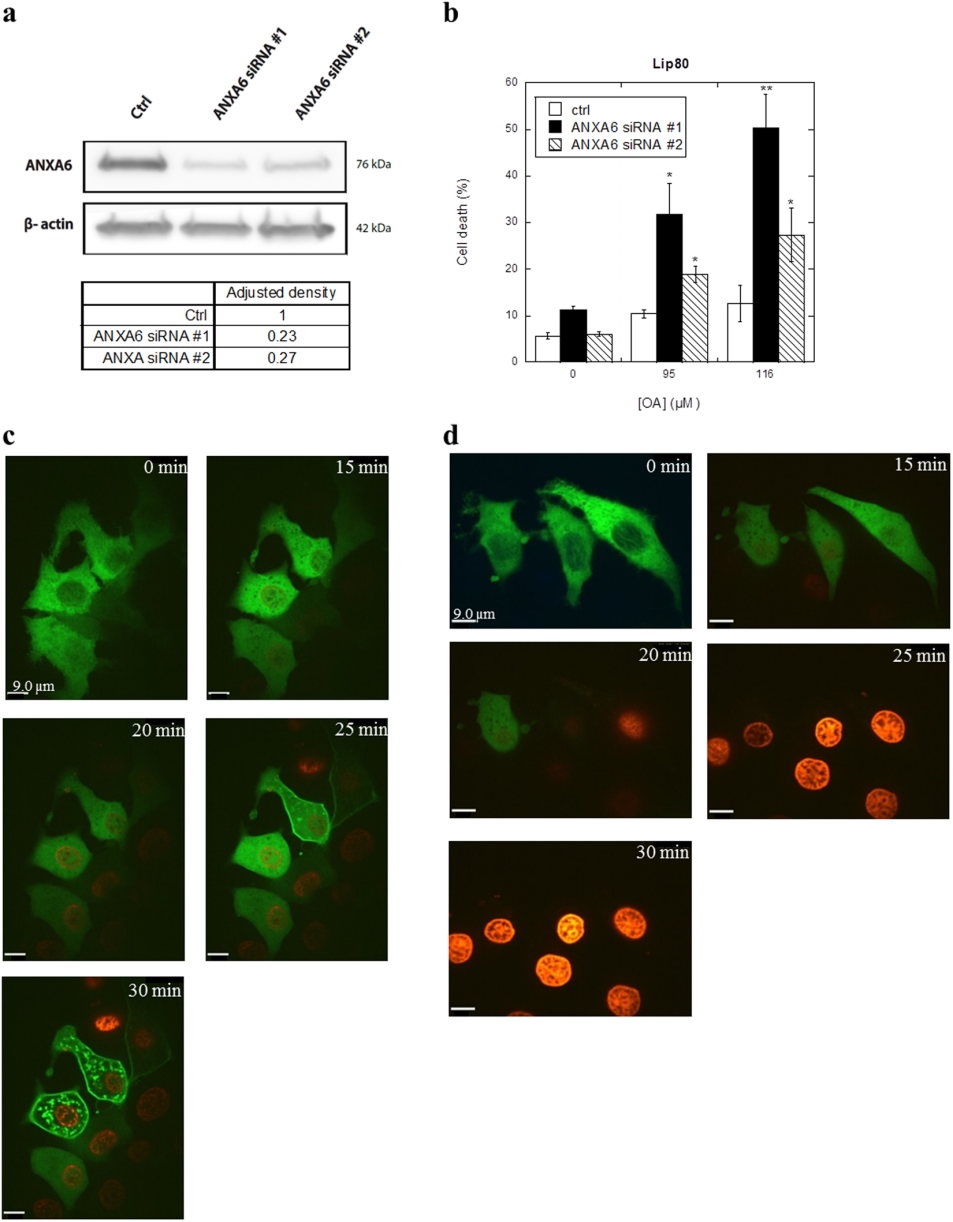
Knockdown of ANXA6 sensitizes to cell death induced by liprotides. (**a**) Immunoblot of ANXA6 protein levels from control and siRNA depleted cells. β-actin was used as control for equal loading. Adjusted density values were calculated by dividing the relative density of the ANXA6 band in each sample lane by the relative density of the β-actin band for the same lane. The lanes are from same gel, but with different exposure times; exposure time for ANXA6 was 120 sec and β-actin 40 sec (Supplementary Fig. S1). (**b**) The percentage of dead cells induced by 0, 95 and 116 µM lip80 plotted as function of OA concentration in lip80. *p < 0.05; **p < 0.01. Panel c and d: Effect of Ca^2+^ on ANXA6-GFP binding to the plasma membrane. (**c**) Cells expressing ANXA6-GFP in media with Ca^2+^ treated with 212 µM lip80. (**d**) Cells expressing ANXA6-GFP in media without Ca^2+^ treated with 212 µM lip80. ANXA6-GFP in green and impermeable Hoechst in blue.

These results inspired us to further examine if the plasma membrane repair system protects against liprotide-induced cell death. As ANXA6 was previously shown to be involved in plasma membrane repair, we depleted ANXA6 with two different siRNAs (#1 and #2) before treating cells with lip80. ANXA6 depletion by both siRNAs reduced ANXA6 protein levels by more than 70% (Fig. 5a; Supplementary Fig. S2) and sensitized MCF7 cells significantly to cell death as compared to cells treated with a control siRNA (Fig. 5b). These data indicate that MCF7 cancer cells are more sensitive to liprotides when membrane repair is compromised through ANXA6, and strongly suggest that liprotides act by disrupting the plasma membrane.

To confirm the involvement of both Ca^2+^ and ANXA6 in the defence against lip80, MCF7 cells expressing ANXA6 fused to GFP (ANXA6-GFP) were imaged by time-lapse microscopy. Treatment of cells in presence of Ca^2+^ induced translocation of ANXA6-GFP to the plasma membrane shortly before membrane permeabilization and cell death, which occurred after 25–30 min of treatment with 212 µM lip80 (Fig. 5c; Supplementary Video S6). Cell death induced by lip80 in the absence of Ca^2+^ was initiated by the disappearance of ANXA6-GFP fluorescence followed by cell death observed after 20 min (Fig. 5d; Supplementary Video S7). Since Ca^2+^ is required for annexin binding to plasma membrane, the disappearance of ANXA6-GFP shows that disruption of the plasma membrane leads to the release of ANXA6-GFP to the extracellular environment. This underpins our earlier results indicating that liprotides disrupts the plasma membrane and that Ca^2+^ and ANXA6 are important for the plasma membrane repair system to counterbalance the effect of liprotides.

### Serum albumin removes OA from liprotides, even when aLA is crosslinked

**Figure 6:**
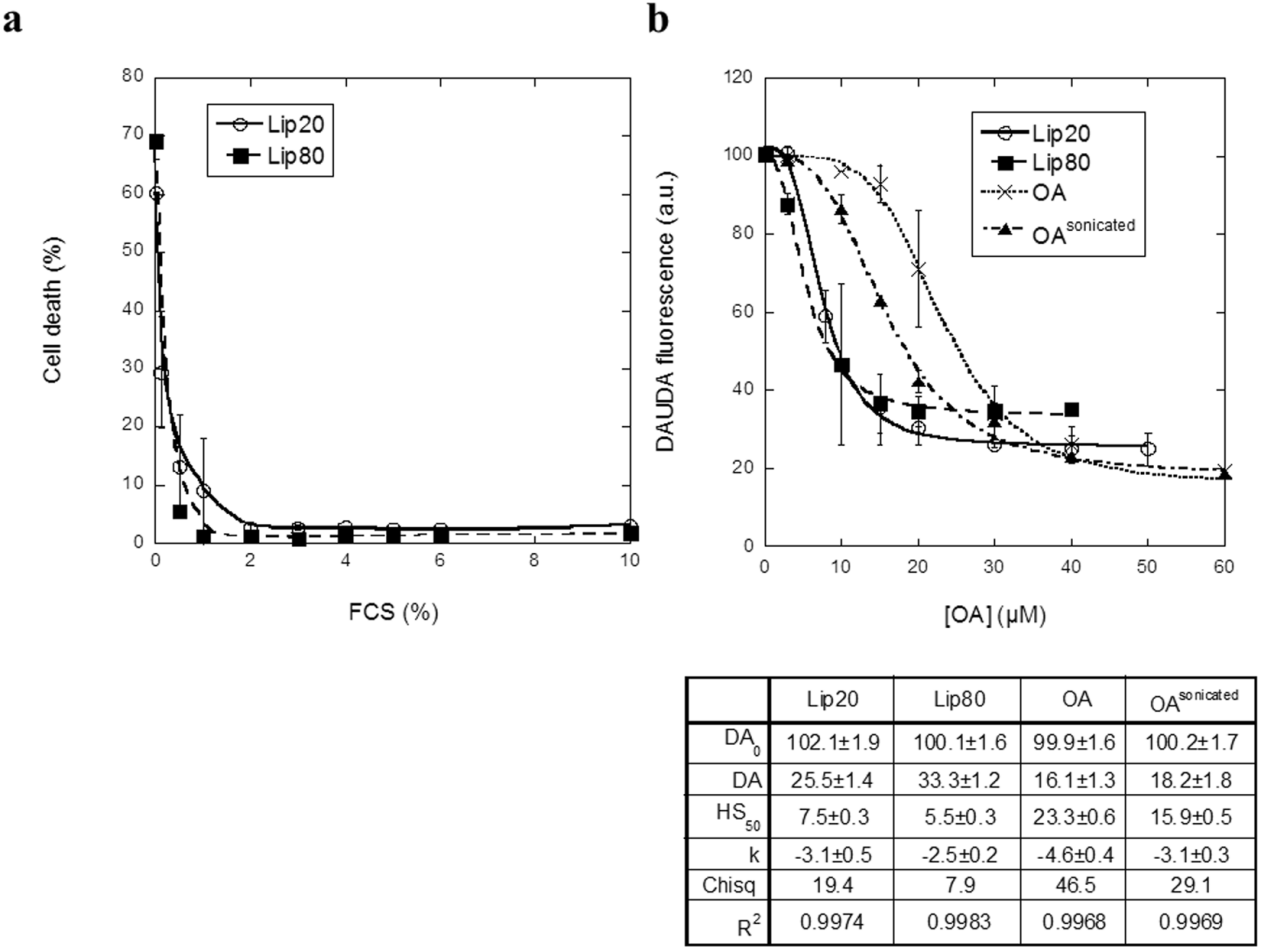
Serum stability of lip20 and lip80. (**a**) Cells treated with 52 µM lip20 and 52 µM lip80 in media with 0–10% FCS. (**b**) BSA complexed with DAUDA was titrated with increasing concentrations of lip20, lip80, OA and OA^sonicated^. A sigmoidal curve (eq. 1) was fitted to data for lip20, lip80 and OA, with fitted parameters indicated below the figure.

Effective systemic application of liprotides for cancer therapy requires good serum stability. The major challenge is that the main protein component of serum is albumin (whose concentration in serum is 35–50 mg/ml^41^) which competes with aLA for OA binding^12^. We therefore tested the toxicity of lip20 and lip80 towards MCF7 cells at different concentrations of FCS. Already at ~1.0% (lip80) and ~2.0% FCS (lip20), the permeabilizing effect decreased to the level of untreated cells (Fig. 6a). Interestingly, lip20 is slightly more robust than lip80, despite lip80′s greater preponderance of intermolecular bonds, in contrast to the situation with Ca^2+^ (Fig. 3). We investigated the role of albumin in removal of OA from liprotides using the environmentally sensitive fluorophore 11-(dansylamino)undecanoic acid (DAUDA) (Supplementary Fig. S3a). DAUDA fluorescence is blue-shifted and increases in intensity upon binding to bovine serum albumin (BSA), and subsequent addition and binding of OA dissociates DAUDA from BSA, leading to a red shift and a decrease in fluorescence (Supplementary Fig. S3b). We measured transfer of OA from liprotides to BSA by measuring the fluorescence intensity at the maximum emission wavelength of DAUDA in complex with BSA (500 nm). Addition of increasing concentrations of lip20 and lip80 results in a decrease of the DAUDA fluorescence signal, indicating that BSA extracts OA from liprotides (Fig. 6b). By fitting a sigmoidal curve (Eq. 1) to the data, we estimated the half saturation concentration (HS_50_) to 7.5 ± 0.3 µM and 5.5 ± 0.3 μM for lip20 and lip80, respectively. This indicates that lip80 is more efficient at transferring its OA to BSA than lip20, consistent with its lower resistance to serum (Fig. 6a). For OA alone and OA sonicated for 6 min (OA^sonicated^), HS_50_ values were 23.3 ± 0.6 µM and 15.9 ± 0.5 μM respectively. OA micelles formed by sonication have been reported by Griebenow and co-workers to have the same cytotoxicity as equimolar concentrations of sonicated OA bound to aLA^42^, most likely because sonication disperses OA more effectively. However, in our hands liprotides are more efficient at solubilizing and presenting OA to BSA than OA or OA^sonicated^, possibly because of differences in the preparation of the complex compared to the Griebenow group^42^.

**Figure 7:**
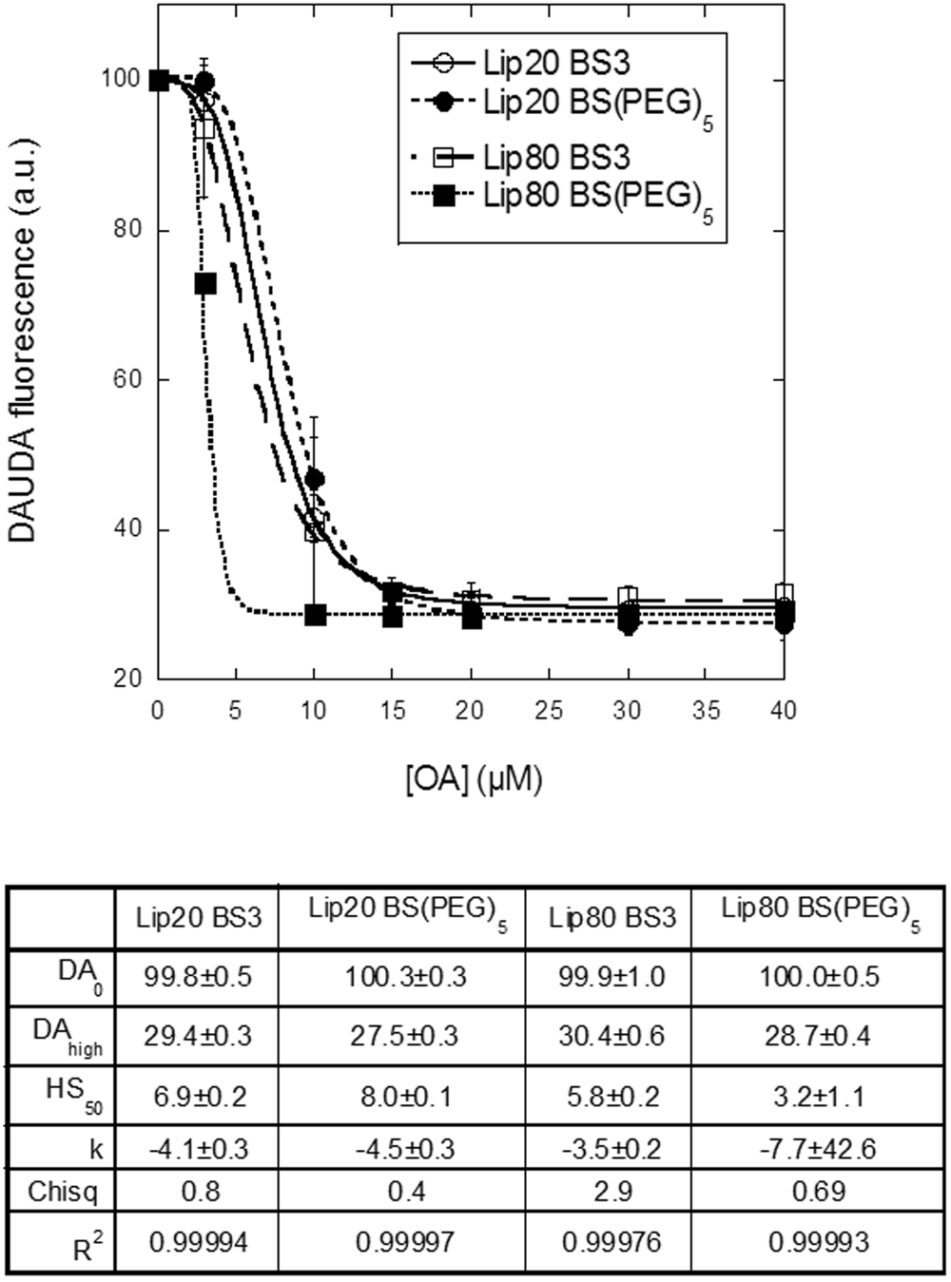
Serum stability of crosslinked lip20 and lip80. BSA complexed with DAUDA was titrated with increasing concentration of lip20 and lip80 crosslinked with BS3 and BS(PEG)_5_. Data fitted as in Fig. 6 and fitted parameters indicated below the figure.

To obtain an independent verdict on the relationship between intermolecular crosslinking and OA release, we analysed whether chemical crosslinking of aLA in liprotides could eliminate or reduce the ability of BSA to extract OA from liprotides. As crosslinkers we used bis(sulfosuccinimidyl)suberate (BS3) and PEGylated bis(sulfosuccinimidyl)suberate (BS(PEG)_5_). Both reagents contain two reactive groups, which form amide bonds with lysine residues (Supplementary Fig. S4). BS3 has a spacer arm length of 11.4 Å, whereas BS(PEG)_5_ has a spacer arm length of 21.7 Å. The crosslinkers were mixed with lip20 and lip80 and the crosslinking was confirmed by SDS-PAGE: A 2–10 fold excess of BS3 over aLA yielded higher order bands and a corresponding reduction in intensity of the monomer band (Supplementary Fig. S5a). The monomer band stabilizes at 35–40% of initial intensity around 30–50 fold excess of BS3. The same trends was shown for lip20 and lip80 crosslinked with BS(PEG)_5_ (data not shown). In both cases we obtained levels of higher order bands comparable with the levels seen in lip80 without crosslinker^22^. Size-exclusion chromatography (SEC) analysis confirmed that crosslinking of liprotide has no impact on the size of the liprotide (Supplementary Fig. S5b). Finally we measured crosslinked liprotides’ ability to transfer OA to BSA using DAUDA fluorescence. The DAUDA fluorescence rapidly decreases with increasing concentration of crosslinked lip20 and lip80 (Fig. 7). This is valid for liprotides crosslinked with BS3 and BS(PEG)_5_. The HS_50_ is 6.9 ± 0.2 µM and 8.0 ± 0.1 μM for lip20 crosslinked with BS3 and BS(PEG)_5_, respectively. For lip80, the corresponding values are 5.8 ± 0.2 µM and 3.2 ± 1.1 µM. Surprisingly, these HS_50_ values are generally lower than lip20 and lip80 without crosslinks, indicating that crosslinking of liprotides makes it easier for BSA to sequester OA from liprotides.

**Figure 8:**
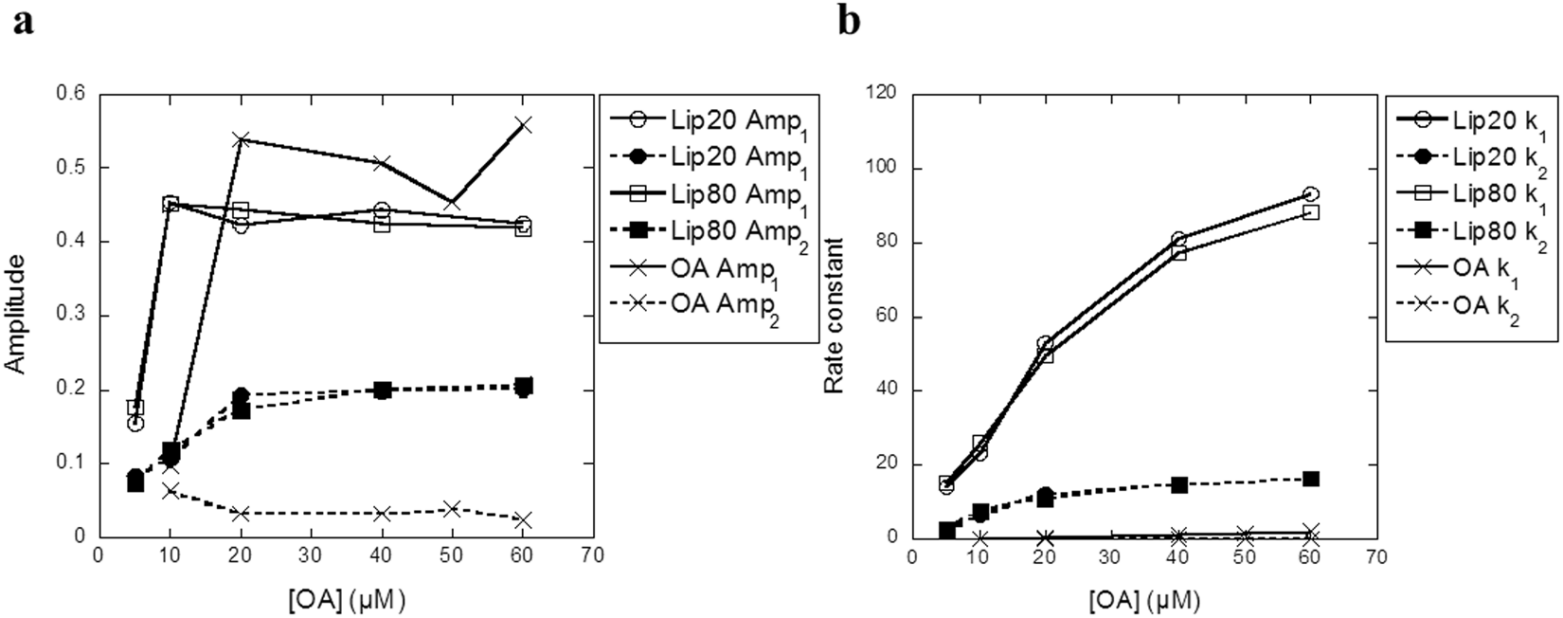
The ability of DAUDA-complexed BSA to remove OA from lip20, lip80 and OA alone was followed by stopped flow and kinetic parameters plotted versus [OA]. (**a**) Amplitudes (Amp) for fluorescence change for lip20, lip80 and OA. (**b**) Rate constants (*k*) for lip20, lip80 and OA.

Finally we used stopped-flow to further investigate the kinetics for the transfer of OA to BSA. In these experiments, one reservoir syringe was filled with BSA bound with DAUDA and the other reservoir syringe was filled with different concentrations of liprotides or OA alone. The contents from the two reservoir syringes were mixed and changes in DAUDA fluorescence signal were measured. For lip20, lip80 and OA a double exponential decay (Eq. 2) fitted the data satisfactorily. Changes in fluorescence amplitude (Amp) and rate constant (k) are plotted as function of OA concentration in Fig. 8. For both lip20 and lip80, a stable amplitude level is reached at 10 µM OA for Amp_1_ and 20 µM OA for Amp_2_, implying that all DAUDA has been displaced from BSA by OA (Fig. 8a). Given that the concentration of DAUDA and BSA is 1.5 µM after mixing, this indicates that DAUDA binds more strongly to BSA than OA does. Both *k*_1_ and *k*_2_ increase with increasing liprotide concentration, indicating that a higher concentration of OA induces a faster displacement reaction (Fig. 8b). For OA alone, a stable amplitude level is reached at 20 µM for both Amp_1_ and Amp_2_, but *k*_1_ and *k*_2_ were much lower than for lip20 and lip80, indicating a slower binding to BSA (Fig. 8a and b). The two different phases of binding to BSA described by the double exponential decay can arise in at least two different ways. For example, DAUDA molecules may bind in two different places on BSA with different affinities, or the replacement of DAUDA with OA is a two-step process and DAUDA fluorescence changes in both steps. The option that OA is released at different rates from liprotides seems less likely, given that the two amplitudes are relatively constant at higher OA concentrations; different rates of release would imply that the faster releasing liprotides should dominate at higher liprotide (and thus OA) concentrations. Further, the intermolecular disulphide bonds in lip80 provide no additional protection against BSA, in agreement with the rest of the data.

BSA stability was also tested for liprotides with a multi-shell structure, giving similar results (data not shown). These data indicate that liprotides are not stable in conditions with serum, and this obviously limits the use of liprotides for cancer treatment.

## Discussion

We find that lip20 and lip80 show similar cytotoxicity towards MCF7, MDA-MB-231 and HeLa cells. This is consistent with reports that liprotides equivalent to HAMLET can kill various cancer cell lines^43–47^. Moreover, the bovine counterpart of BAMLET shows similar cytotoxicity against a broad range of different cancer cell lines^14^.

Investigation of the rate of induced cell death indicates that lip20 induces cell death more rapidly than lip80. Our previous SAXS studies indicated that lip20 and lip80 have similar overall structure^40^. However, high-temperature preparation methods can induce disulphide bond shuffling as is the case for lip80^22^. The lower rate of induced cell death for lip80 may therefore be ascribed to intermolecular disulphide bonds. Unfortunately, neither intermolecular disulphide bonds nor crosslinking with BS3 or BS(PEG)_5_ provides protection against extraction by serum albumin. However, the ability to transfer OA to membranes appears to be critical to liprotide function, so it is entirely to be expected that proteins such as BSA with several high-affinity hydrophobic binding sites are able to extract OA from liprotides. It may be envisaged that the intermolecular disulphide bonds in lip80 restrict aLA in a locked conformation whereas aLA in lip20 is more dynamic and flexible. Apparently this increased flexibility gives higher protection against serum albumin, and in addition facilitates efficient delivery of OA to the plasma membrane. The reason for these opposing effects is unclear, but flexibility may give a better ability to accommodate the OA micelle and thus leads to a higher OA affinity for lip20 compared to lip80 when exposed to BSA, while the flexibility may also make it easier to interact with the hydrophobic cell membrane and thus release bound OA. In contrast, the restricted structure of lip80 may make it more difficult to adjust to OA micelles (and thus compete with BSA), and at the same time also reduce interactions with the membrane. In spite of low serum albumin stability, these results indicate a higher Ca^2+^ stability than predicted for lip20. Ca^2+^ increases lip20′s LD_50_ value of lip20 from 30.2 ± 0.5 µM to 80.8 ± 6.3 µM, whereas that of lip80 is only increased to 48.9 ± 0.5 µM. This indicates that lip20 is less stable and effective in media with Ca^2+^ compared to lip80. Our earlier *in vitro* study showed that a 10-fold excess of Ca^2+^ led to complete refolding of aLA in lip20^40^. However, in the cell culture media with Ca^2+^, a Ca^2+^ concentration higher than 10-fold excess was used (molar ratio > 60). This may be caused by the fact that the cell media is a complex solution with many molecules and salt components, several of which can bind Ca^2+^, making the absolute concentration of free Ca^2+^ unknown.

Cell death induced by liprotides is rapid, occurring within an hour. Lethal changes in transcription, translation or the cell-deviation cycle are slower processes and can therefore be ruled out as key targets for the liprotide. The rapid induced cell death is therefore in good agreement with our suggested cell death mechanism for liprotides. Removal of Ca^2+^ from the cell media increases the number of dead cells, increases the rate of plasma membrane injury and prevents ANXA6-GFP plasma membrane binding. Together with observations of cytosolic GFP leaking to the extracellular environment after treatment with liprotides and higher sensitivity against liprotides for cells with ANXA6 knock down, this suggests that liprotides disrupt the plasma membrane. As OA is a hydrophobic compound, it is favourable for OA to bind to membranes. Thanks to its *cis* double bond, OA has a kinked structure, so that transfer of OA to the plasma membrane will increase membrane disorder. Delivery of liprotides with a core of 12–33 OA molecules to a single area of the plasma membrane could possible destabilize and damage the membrane in a cumulative fashion, eventually leading to breaching of the membrane and cell death.

HAMLET has been shown to interact with different parts of the cell e.g. mitochondria, proteasomes and histones as mentioned in the introduction, probably due to internalization of the complexes by endocytic mechanisms and by gradual plasma membrane permeabilization. Our data indicate that disruption of the plasma membrane is a major factor in liprotide toxicity towards cancer cells.

## Material and Methods

### Cell culture

Cell lines with well-defined genetic status were obtained from ATCC (MCF7: https://www.atcc.org/Products/All/HTB-22.aspx. MDA-MB-231: https://www.atcc.org/Products/All/HTB-26.aspx#generalinformation. HeLa: https://www.atcc.org/Products/All/CCL-2.aspx.). Cells were cultured in RPMI medium (1640) (Gibco by Life Technologies (Carlsbad, CA, USA)) supplemented with 0.25% Pen/Strep (Gibco, 15140-122) and heat-inactivated 6% fetal calf serum (FCB) (Biological Industries) and kept at 37 °C in a humidified atmosphere of 5% CO_2_.

### Plasmid constructs and siRNAs

#### Plasmids

Cells were transfected with a plasmid containing turbo green fluorescence protein (GFP) (Origene (Rockville, MD, USA), PS100010) or corresponding plasmid containing C-terminally tagged ANXA6 transcript variant 1 (Origene, RG202086) using Opti-MEM® (Life Technologies, 11058-021) and Lipofectamine LTX reagent (Life Technologies, 15338-100) (DNA:LTX ratio was 1:4).

#### siRNAs

Sequence of the siRNAs used is ANXA6#1:5′-GACUUAAUGACUGACCUGA[dT][dT]-′3, ANXA6#2: 5′-CCUAUUGUGAUGCCAAAGA[dT][dT]-′3 (Sigma-Aldrich (St. Louis, MO, USA)). Control siRNA: AllStar Negative Control siRNA (QIAGEN (Hilden, Germany)). All transfections were performed using Oligofectamine™ Transfection Reagent (Invitrogen (Carlsbad, CA, USA), 12252-011) with a siRNA concentration of 20 nM. Knockdown was analyzed after 72 h.

### Immunoblot analysis

Protein lysates were separated by SDS–PAGE (Bio-Rad (Hercules, CA, USA), 4–15% Mini-PROTEAN® TGX™ Precast Protein Gels, 4561083) and transferred to 0.2 μm nitrocellulose membranes (Bio-Rad, Trans-Blot® Turbo™ Mini Nitrocellulose Transfer Packs, 1704158). The membranes were incubated overnight in primary antibodies raised against human Annexin A6 (1:1000) (Abcam (Cambridge, UK), ab31026) and β-actin (1:5000) (Sigma-Aldrich, A2228) followed 1/2 h incubation by the appropriate peroxidase-conjugated secondary antibodies (DAKO (Glostrup, Denmark)). Blots were developed using Clarity™ Western ECL Substrate (Bio-Rad) and images were acquired using Luminescent Image Analyzer LAS-4000 mini (Fujifilm). Quantification was performed in ImageJ.

### Production of liprotides

Liprotides were routinely formed by mixing calcium depleted α-lactalbumin from bovine milk (aLA, ≥85%, Sigma-Aldrich, L6010) dissolved in Milli-Q water and 125 mM sodium oleate (OA, ≥95% pure, Sigma-Aldrich, O3880) dissolved in 20% ethanol at an aLA:OA molar ratio of 1:15 in 50 mM PBS (50 mM NaHPO4, pH 7.4, 150 mM NaCl) and incubated for 1 h at 20 °C or 80 °C (leading to lip20 and lip80 respectively). These conditions allow OA to form complexes with aLA which adopt a core-shell structure according to previous Small Angle X-ray Scattering studies^40^, in which the core is largely made of OA assembled in a micelle and the shell contains partially denatured aLA molecules.

### Cell Death Assays

#### PI exclusion assay

Cell death and PI kinetics were measured using the Celígo® Imaging Cytometer (Brooks Life Science Systems). The cells were cultured with RPMI serum free medium just before treatment with lip20, lip80, OA, aLA20 or aLA80 in PBS (DPBS, -CaCl_2_, -MgCl_2_ (Life Technologies, 14190-094)). For experiments investigating the effect of serum, cells were cultured with 0.1–10% FCS. When the Ca^2+^ effect was investigated, cells were cultured in HBSS medium with or without CaCl_2_ and MgCl_2_ (Gibco, 14025 or 14175). For both cell death and PI kinetic measurements, cells were stained with 5 μg/ml permeable Hoechst-33342 (Sigma-Aldrich, B2261) and 0.1μg/ml PI solution ( ≥94% pure, Sigma-Aldrich, P4864) for 5 min at 37 °C. The PI fluorescence intensity (integrated) was measured at different time points and cell death was measured after 1 h at 37 °C. Cell death [%] was plotted against OA concentration for OA and liprotides. Data are based on triplicate or quadruplicate measurements. A sigmoidal curve (eq. 1) was fitted to the data using KaleidaGraph version 4.0 (Synergy) when possible:

1 $$CD=C{D}_{0}+(95-C{D}_{0})/(1+{(L{D}_{50}/[OA])}^{k})$$

where CD is the measured cell death in percent, CD_0_ is cell death at 0 µM OA, LD_50_ is the dose causing 50% cell death and *k* is a constant describing the cooperativity of the OA effect, *i.e*. the steepness of the transition. The term “95-CD_0_” is the amplitude of the signal change between low and high [OA], given that we only observe up to 95% cell death.

#### Hoechst-33258 cell death assay and membrane integrity assay

To measure membrane integrity and cell death, cells were incubated with 2 µM FM1-43 dye (Life Technologies, T3163) and 2 µg/ml impermeable Hoechst-33258 (Sigma-Aldrich, 861405) before treatment with liprotide or PBS. Cell were imaged every 15 seconds on a Carl Zeiss Axiovert 200 M fluorescence time-lapse microscope, 20x objective and analysed using MetaMorph software.

### Imaging of GFP and ANXA6-GFP translocation

Translocation of GFP and ANXA6-GFP in cells in the presence and absence of Ca^2+^ was investigated with Nicon confocal spinning disk (PerkinElmer) microscope through a 63x objective.

### 11-(Dansylamino)undecanoic Acid (DAUDA) fluorescence

11-(dansylamino)undecanoic acid (DAUDA, Sigma-Aldrich, 39235) fluorescence was measured with Cary Eclipse fluorescence spectrometer (Agilent Technologies, Santa Clara, CA, USA) with excitation at 335 nm and emission at 500 nm. Spectra were recorded with slit widths of 5 nm at room temperature (RT). 1 ml sample was prepared containing 3 μM essentially fatty acid free bovine serum albumin (BSA, ≥96% pure, Sigma-Aldrich, 3912) in complex with 3 μM DAUDA. After reaching a stable fluorescence level, it was mixed with increasing concentrations (3–120 μM in terms of OA concentration) of liprotide, OA or OA^sonicated^ (OA sonicated 6 min at 20% of max power with QSONICA sonicator (Newtown, CT)). Data were fitted to the sigmoidal curve used in eq. 1, except that CD is replaced by *F* (measured DAUDA fluorescence), 95 by DA_high_ (fluorescence at high [OA]), CD_0_ by DA_0_ (fluorescence at 0 µM OA) and LD_50_ with HS_50_ (half saturation concentration).

### Crosslinking

aLA in liprotides was crosslinked with bis(sulfosuccinimidyl)suberate (BS3, Sigma-Aldrich, 21580) and PEGylated bis(sulfosuccinimidyl)suberate (BS(PEG)_5_, Sigma-Aldrich, 21581). The crosslinkers were dissolved in Milli-Q water to a concentration of 100 mM and then diluted to 12.5 mM in 50 mM PBS (50 mM NaHPO_4_, pH 7.4, 150 mM NaCl). For SDS-PAGE (15% SDS-PAGE gel), crosslinkers were mixed at 2–50 fold excess with 1 mg/ml liprotide and incubated at RT for 30 min. The crosslinking reaction was stopped by adding Tris to 1 M. For size exclusion chromatography (SEC) and measurements of DAUDA fluorescence, crosslinkers were mixed at 30-fold excess with 2 mg/ml liprotide at RT and incubated for 30 min, after which the crosslinking reaction was stopped with 1 M Tris. Sample was injected through a 250 µl sample loop onto a Superdex 200 Increase column (GE Healthcare, Little Chalfort, UK), connected to a Äkta Pure system (GE Healthcare). The flow rate was 0.5 ml/min and absorption was measured at 280 nm.

### Stopped flow

Stopped-flow experiments were carried out on an SX18.MV rapid-reaction microanalyzer (Applied Photophysics (Surrey, UK)) at RT. BSA’s ability to capture OA from liprotides were followed via the displacement of bound DAUDA, which in turn was monitored by exciting at 335 nm and measuring emission with a 435 nm cut-off filter. Data were analyzed using the software provided by the manufacturers. 3 μM BSA in an equimolar complex with DAUDA was mixed 1:1 with different concentrations of liprotide and OA (5–10 μM), giving a final concentration of 1.5 μM BSA after mixing. Data were fitted to a double exponential decay function:

2 $$F=Am{p}_{1}\ast {e}^{-{k}_{1}t}+Am{p}_{2}\ast {e}^{-{k}_{2}t}$$

where *F* is the fluorescence change measured, *Amp*_x_ is amplitude, *k*_x_ is a rate constant and *t* is time.

### Statistical analysis

A two-tailed *t*-test was used to determine if the percentage cell death induced by lip80 was significantly different for cells with ANXA6 knockdown compared to control cells.

## Electronic supplementary material

Video S1

Video S2

Video S3

Video S4

Video S5

Video S6

Video S7

Supplementary information
