# Supplementary material for: Liprotides kill cancer cells by disrupting the plasma membrane
Source: Sci Rep. 2017 Nov 9;7:15129. doi: 10.1038/s41598-017-15003-6 (PMC5680231; doi:10.1038/s41598-017-15003-6)
Supplement: Supplementary file 8 — Supplementary information [file 41598_2017_15003_MOESM8_ESM.pdf]

Liprotides kill cancer cells by disrupting the plasma membrane

Henriette S. Frislev, Theresa Louise Boye, Jesper Nylandsted and Daniel Otzen

Supplementary Figures and Videos

a **b**

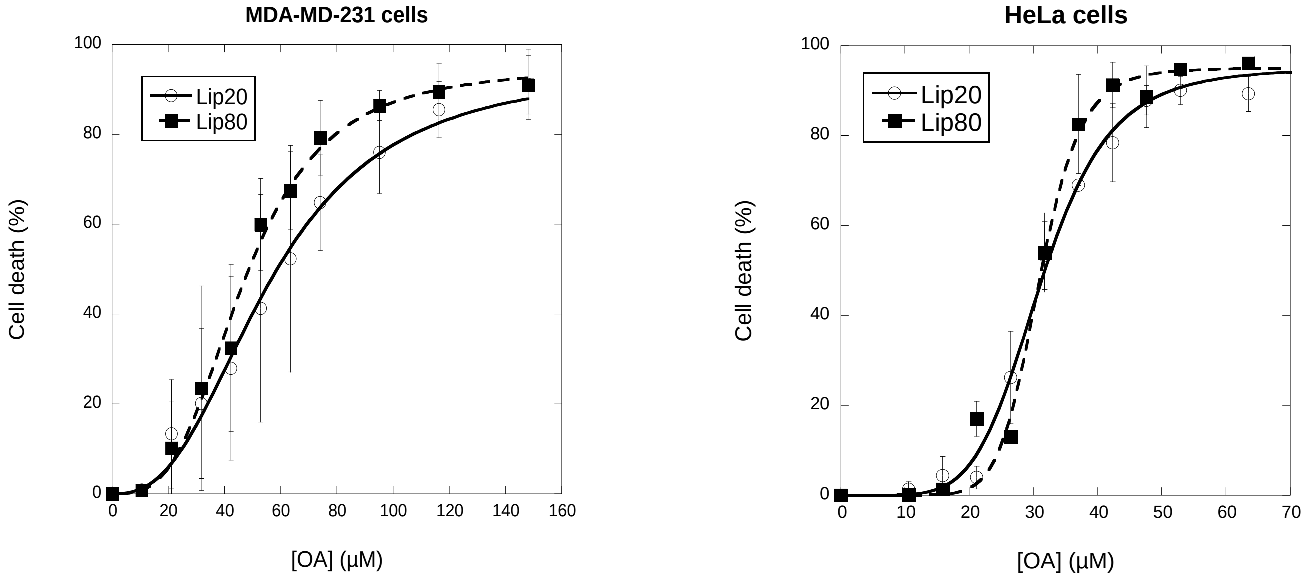

(a)

	Lip20	Lip80
CD_0	0±3.4	0±3.3
LD_{50}	47.1±1.1	56.4±1.4
k	3.2±0.2	2.6±0.2
Chisq	90.3	87.6
R^2	0.9932	0.9921

(b)

	Lip20	Lip80
CD_0	0±2.8	0±5.4
LD_{50}	31.2±0.4	30.9±
k	-5.7±0.4	-9.5±1.5
Chisq	71.45	263.5
R^2	0.9979	0.9935

Figure S1: Percentage dead cells plotted versus OA concentration. (a) MDA-MD-231 cells treated with lip20 and lip80. (b) HeLa cells treated with lip20 and lip80. A sigmoidal curve (Eq. 1) was fitted to data for lip20 and lip80 with fitted parameters indicated below the figures.

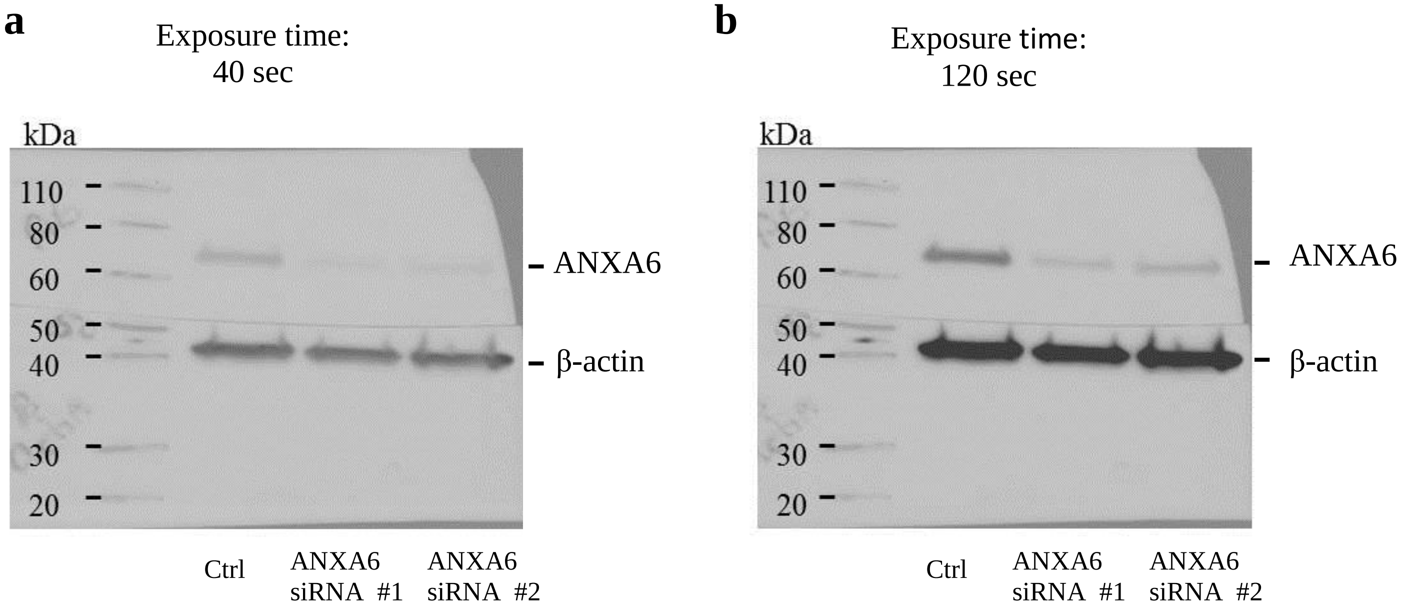

Figure S2: Immunoblot of ANXA6 protein levels from control and siRNA depleted cells. β-actin was used as control for equal loading. (a) Immunoblot achieved by exposure time of 40 sec. (b) Immunoblot achieved by exposure time of 120 sec.

a **b**

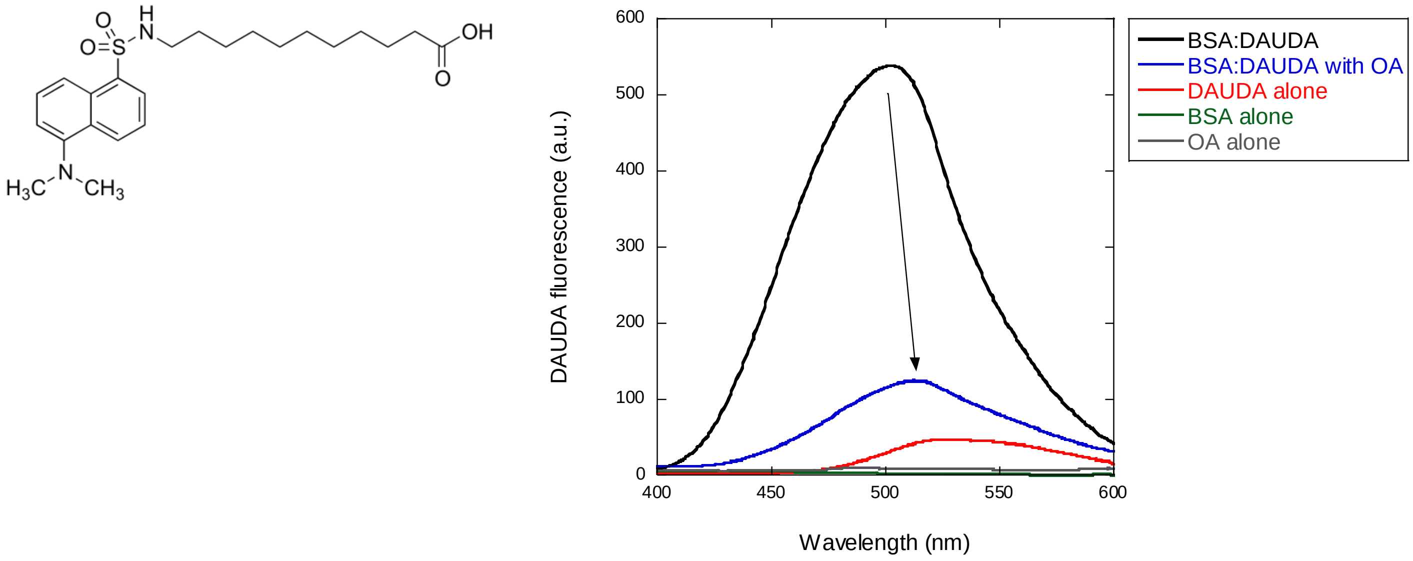

Figure S3: Change in DAUDA fluorescence cause by OA. (a) Structure of DAUDA. (b) DAUDA fluorescence measured for DAUDA in complex with BSA (BSA:DAUDA) and mixed with OA and control measurements with DAUDA alone, BSA alone and OA alone. Adding OA to a sample with BSA:DAUDA decreases and red-shifts the fluorescence of DAUDA.

a **b**

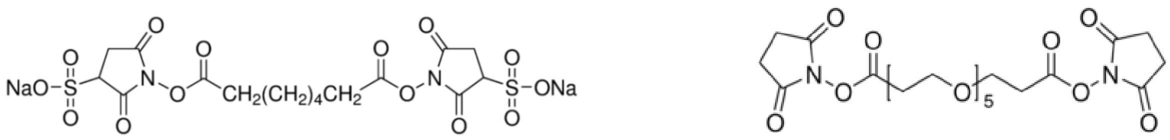

BS3 $BS(PEG)_5$

Figure S4: Structure of crosslinkers. (a) BS3. (b) $BS(PEG)_5$.

a

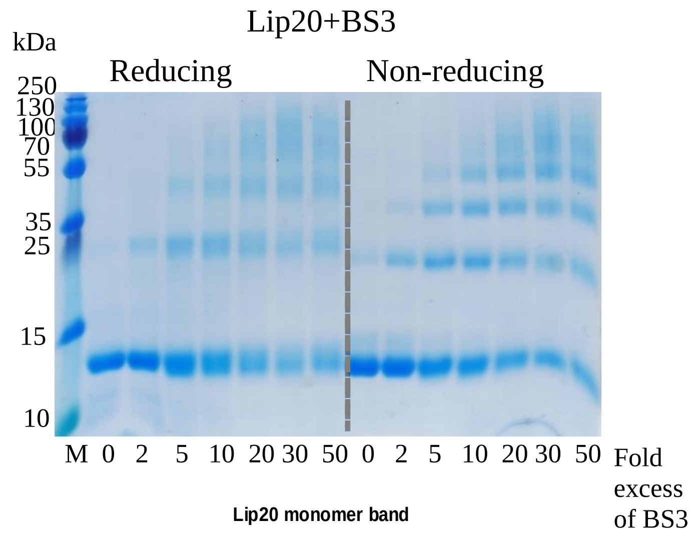

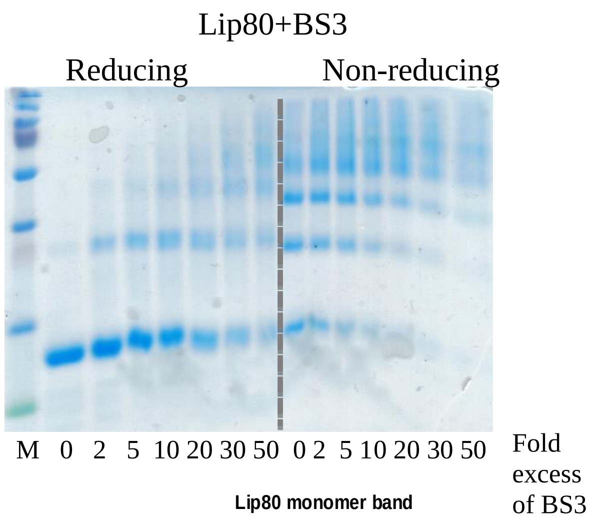

Lip20 monomer band

Fold excess of BS3	Relative density Reducing	Relative density Non-reducing	Relative difference [Reducing/Non-reducing]*100
1	1,00	1,00	100
2	0,93	1,00	93
5	1,04	0,83	125
10	0,84	0,66	127
20	0,52	0,44	117
30	0,35	0,32	108
50	0,39	0,30	128

Lip80 monomer band

Fold excess of BS3	Relative density Reducing	Relative density Non-reducing	Relative difference [Reducing/Non-reducing]*100
1	1,00	1,00	100
2	0,96	0,97	99
5	0,87	0,69	125
10	0,94	0,60	157
20	0,53	0,40	132
30	0,33	0,11	304
50	0,28	0,09	310

b

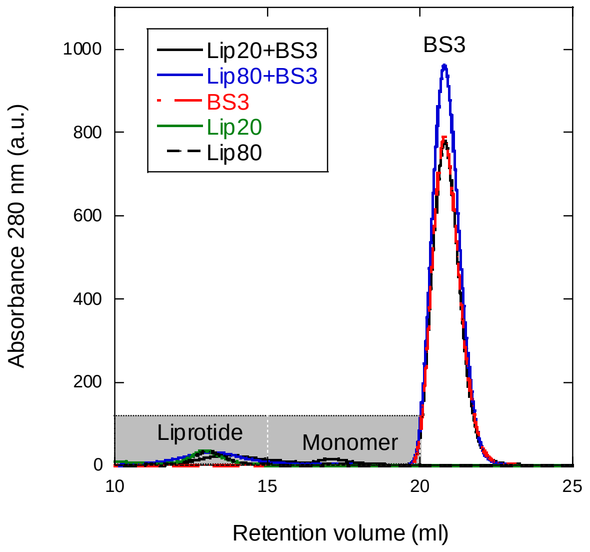

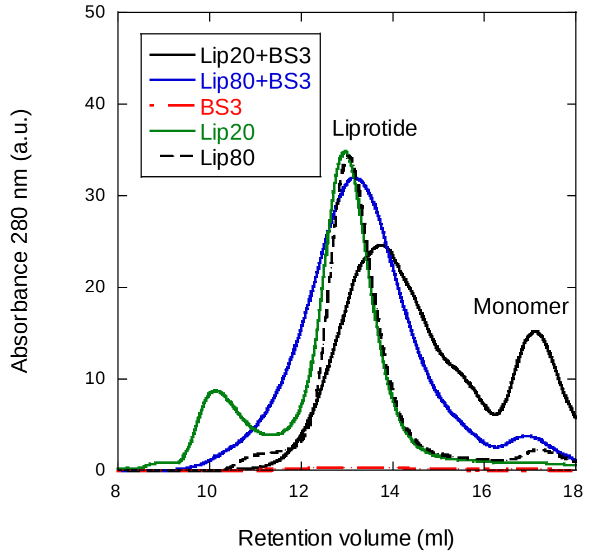

Figure S5: Lip20 and lip80 crosslinked with BS3. (a) SDS-PAGE of lip20 and lip80 crosslinked with BS3 in non-reducing and reducing conditions. The relative monomer band density is shown in the table below the figure. (b) SEC chromatogram of lip20 and lip80 crosslinked with BS3 (left) and a close up of the gray box (right).

LEGENDS FOR SUPPLEMENTARY VIDEOS

Video S1 (file name S1.lip20): MCF7 cells treated with 52 µM lip20 at time 0 min and induced cell death followed by FM1-43 shown in green and impermeable Hoechst in red.

Video S2 (file name S2.lip80): MCF7 cells treated with 52 µM lip80 at time 0 min and induced cell death followed by FM1-43 shown in green and impermeable Hoechst in red.

Video S3 (file name S3.lip80GFP): MCF7 cells expressing GFP treated with 212 µM lip80 at time 0 min. GFP is shown in green and impermeable Hoechst in red.

Video S4 (file name S4.lip80-ca): MCF7 cells in media without Ca^{2+} treated with 52 µM lip80 at time 0 min and induced cell death followed by FM1-43 shown in green and impermeable Hoechst in red.

Video S5 (file name S5.lip80+ca): MCF7 cells in media with Ca^{2+} treated with 52 µM lip80 at time 0 min and induced cell death followed by FM1-43 shown in green and impermeable Hoechst in red.

Video S6 (file name S6.lip80+ca): MCF7 cells expressing ANXA6-GFP in media with Ca^{2+} treated with 212 µM lip80 at time 0 min. Green is ANXA6-GFP and blue is impermeable Hoechst.

Video S7 (file name S7.lip80-ca): MCF7 cells expressing ANXA6-GFP in media without Ca^{2+} treated with 212 µM lip80 at time 0 min. Green is ANXA6-GFP and blue is impermeable Hoechst.
